# Time-efficient fabrication method for 3D-printed microfluidic devices

**DOI:** 10.1038/s41598-022-05350-4

**Published:** 2022-01-24

**Authors:** Yan Jin, Peng Xiong, Tongyu Xu, Jingyi Wang

**Affiliations:** 1 College of Information and Electrical Engineering, Shenyang Agricultural University, Shenyang, 110866 China; 2 Liaoning Engineering Research Center for Information Technology in Agriculture, Shenyang, 110866 China

**Keywords:** Nanofluidics, Fluidics

## Abstract

Recent developments in 3D-printing technology have provided a time-efficient and inexpensive alternative to the fabrication of microfluidic devices. At present, 3D-printed microfluidic systems face the challenges of post-processing, non-transparency, and being time consuming, limiting their practical application. In this study, a time-efficient and inexpensive fabrication method was developed for 3D-printed microfluidic devices. The material for 3D-printed microfluidic chips is Dowsil 732, which is used as a sealant or encapsulant in various industries. The curing time and surface hydrophobicity of the materials were evaluated. The results indicated that the surface of Dowsil 732 is hydrophilic. An optimization model of the direct ink writing method is proposed to establish a time-efficient and accurate fabrication method for microfluidic devices. The results indicate that the optimization model can effectively describe the change trend between printing speed, printing pressure, and channel wall accuracy, and the model accuracy rate exceeds 95%. Three examples—a micromixer, concentration gradient generator, and droplet generator—were printed to demonstrate the functionality and feasibility of the fabrication method.

## Introduction

Microfluidic equipment^1^ has the characteristics of small size, low cost, fast response, and high detection sensitivity^2^, and it has been widely used in many fields, such as biomedicine^3,4^, chemical synthesis^5^, agricultural governance^6^, and environmental testing^7^. In recent years, with the rapid development of modern additive manufacturing techniques, three-dimensional (3D)-printing technology has become a promising method for microfluidic device fabrication. Compared with traditional micro processing technologies, such as soft lithography^8^, computer numerical control milling^9,10^, laser cutting^11,12^, and injection molding^13^, 3D-printing technology has the advantages of rapid manufacturing^14^, wide material adaptability^15^, and low cost^16^. The 3D-printing technology provides a potential low-cost and time-saving alternative to conventional polydimethylsiloxane (PDMS) microfluidic systems, simplifies the traditional manual fabrication process, and reduces the need for professional microfabrication^17^.

At present, the best candidates or microfluidic devices through 3D-printing technology are stereolithography (SLA)^18,19^, digital light projection (DLP)^20,21^, fused deposition modeling (FDM)^22–24^, and direct ink writing (DIW)^25,26^. SLA and DLP are based on the selective curing of a photosensitive polymer to print the desired structures. The microfluidic devices produced by SLA printing technology have the advantage of high precision^27,28^, but the photosensitive resin materials may remain in the micro channel, causing blockage inside the channel^29^. In addition, the preprocessing and post-processing of microfluidic devices can result in a relatively complicated and time-consuming manufacturing process^30^. The FDM or DIW printing technology is mainly based on the material extrusion method, and the fabrication process is relatively accessible. The printers for the FDM method are much more accessible because of their low prices. Compared with the SLA or DLP methods, the FDM or DIW method provides a wider material selection^31–33^. Biocompatible and inexpensive polymer materials, such as poly lactic acid^34,35^, acrylonitrile butadiene styrene^36,37^, and NinjaFlex (flexible material)^38,39^ make the FDM method an ideal candidate for 3D printing of microfluidic devices. However, most microfluidic devices using the FDM method are nontransparent or semitransparent, making them unsuitable for observation or optical detection.

Here, a time-efficient, inexpensive DIW method is proposed for the 3D printing of microfluidic devices. A microfluidic chip with a complex structure can be manufactured within one hour. The material for 3D-printed microfluidic chips is Dowsil 732 from Dow Corning (Midland, MI, USA). Similar to PDMS, Dowsil 732 is used as a sealant or encapsulant in many industries. However, no research related to the Dowsil 732 microfluidic device has been reported. In this work, first, the curing time and surface hydrophobicity of Dowsil 732 were evaluated, proving its suitability for the fabrication of microfluidic devices. To ensure that a high-precision micro channel structure can be obtained, the influence of printing pressure and printing speed on the accuracy of the channel wall were investigated, and a printing parameter optimization model was established based on measured data. Then, the accuracy between the micro channel design size and the actual printed size was examined further. Finally, three printing examples (a micro mixer, concentration gradient generator, and droplet generator) were used to verify the feasibility of the research theory.

## Results and discussion

### Dowsil 732 properties for microfluidic devices

**Figure 1:**
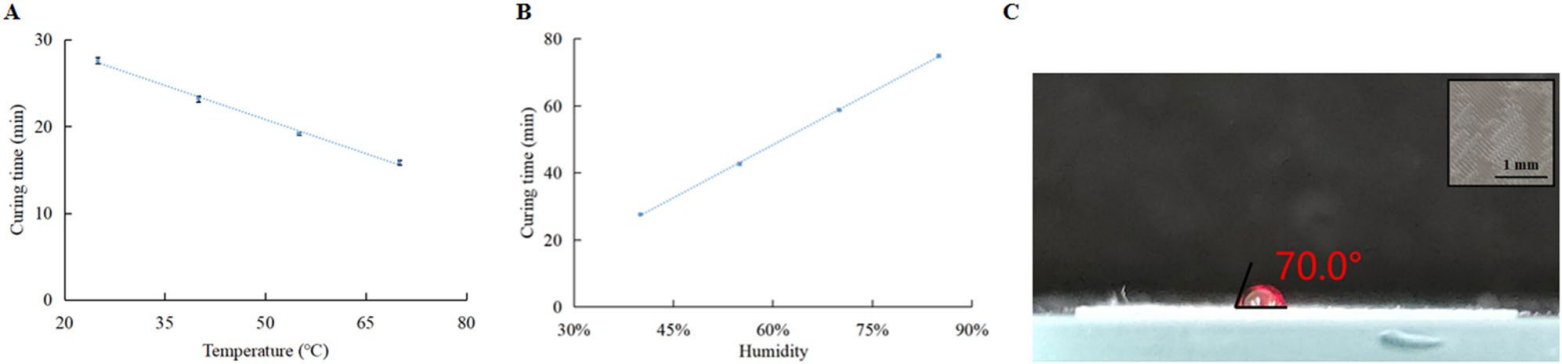
(**A**) The curing time of Dowsil 732 at different temperature and (**B**) the curing time of Dowsil 732 at different humidity (each curing time is measured three times for every temperature and humidity), (**C**) contact angle measurement for Dowsil 732.

A series of calibration tests were conducted to determine the optimized parameters for microfluidic devices based on Dowsil 732. More specifically, the curing time, transparency and hydrophilicity of Dowsil 732 were investigated. In this work, only the Dowsil 732 material was used to fabricate the micro channel wall structure, and the substrate and cover plate of the microfluidic devices were made of highly transparent acrylic plates, so that the fluids inside the microfluidic device could be directly observed by the naked eye or optical instruments. Hence, a Dowsil 732 sheet was printed and fabricated for the curing time and hydrophilicity testing. The simple variable method is introduced in the test to find the best curing time. The curing time test with constant humidity (40%) is shown in Fig. 1A. The curing time decreases with the increase of the temperature due to the accelerated reaction of Acetoxy cure process. Figure 1B shows that the curing time increases from 27.5 to 75 min when the humidity value changes from 40 to 85%, meaning that the higher humidity environment inhibits the curing efficiency of the Dowsil 732. Thus, the best curing condition under comprehensive consideration is as follow: 70 °C and 40% relative humidity. When the humidity changes, the temperature was maintained at 70 °C to ensure that the Dowsil 732 can cure quickly. In addition, the contact angle of the red dye droplet on the Dowsil 732 surface was measured, as depicted in Fig. 1C. Measuring this parameter revealed that Dowsil 732 has a smaller contact angle, which means that Dowsil 732 is a hydrophilic material (more wettable), which is an essential feature for microfluidic devices.

### Optimization model of printing parameters

**Figure 2:**
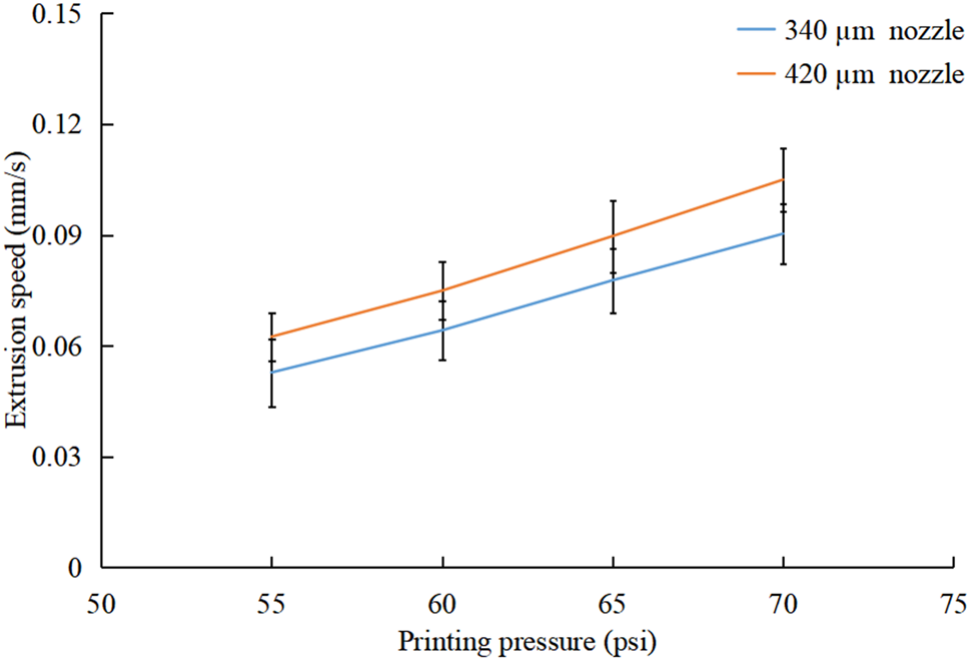
Relationship between printing pressure and extrusion speed of Dowsil 732: each extrusion speed was recorded five times for every pressure.

The printing pressure is one of the main characteristics for controlling the extrusion speed of Dowsil 732 and the printed width of the microchannel wall. To print the microfluidic devices accurately, the extrusion speeds of the 340 and 420 µm nozzles were measured in the pressure range of 55–70 psi (the nozzle remained stationary). The measurement indicated that the extrusion speed of Dowsil 732 progressively increased as the printing pressure increased for different nozzles (Fig. 2). The extrusion speed difference between the two nozzles at the same pressure is 0.01 mm/s.

**Figure 3:**
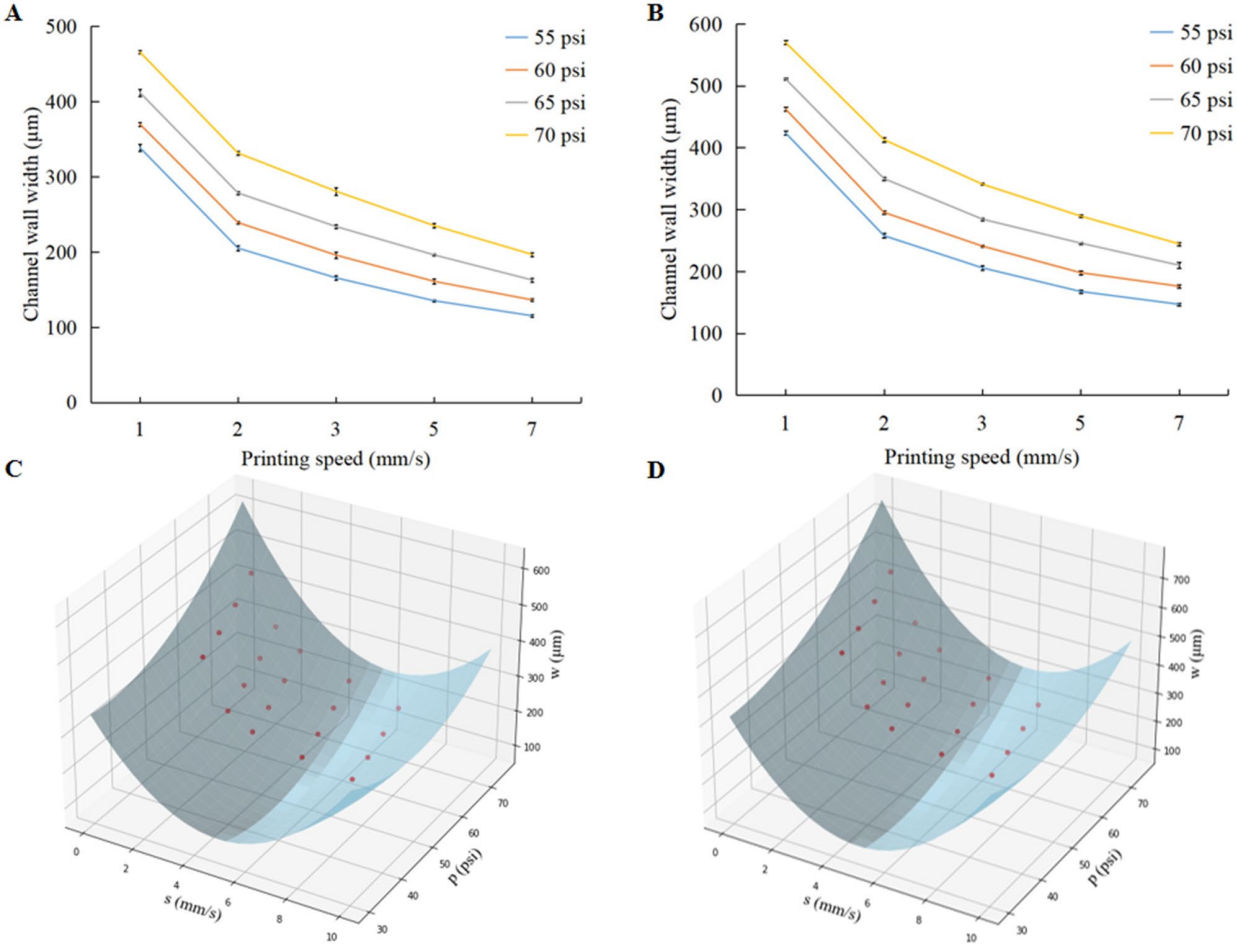
(**A**) Plot showing the variation trend of channel wall width under different printing speed and printing pressure by a 340 µm nozzle, and (**B**) plot showing the variation trend of channel wall width under different printing speed and printing pressure by a 420 µm nozzle (each channel wall width is measured three times for every pressure and speed). (**C**) Plot showing the surface fitting of experimental data of a 340 µm nozzle, and (**D**) plot showing the surface fitting of experimental data of a 420 µm nozzle.

In addition, the print accuracy of the channel wall is influenced by the printing speed (moving speed of the nozzle), printing pressure, and lateral diffusion of the printing materials. The variation in the channel wall width at different printing speeds and printing pressures was then investigated. Figure 3A, B indicate that the variation trend of the channel wall width is similar between the two types of nozzle. Figure 3A, B reveal that, with the same printing pressure, the channel wall width gradually decreases as the printing speed increases. In contrast, at the same printing speed, there is a positive correlation between the channel wall width and printing pressure. Based on the image analysis, the curved surface equation consisting of the printing speed and printing pressure can be used to describe the channel wall widths. Therefore, the experimental data obtained for the two nozzles were fitted on different surfaces using second-order polynomials. The models were developed for 340 and 420 µm nozzles. Polynomial equations were obtained for the printed width of the channel walls of the two nozzles. The model equations are

1 $$ w_{340} = 9.72s^{2} - 0.525sp + 0.168p^{2} - 80.5s - 11.9p + 558.2$$

2 $$ w_{420} = 12.56s^{2} - 0.584sp + 0.171p^{2} - 107.5s - 10.6p + 584.5 $$

where *s* and *p* denote the print speed and print pressure, respectively, and *w* denotes the microchannel wall width. Plots describing the surface fit of the experimental data with the modeled surfaces are shown in Fig. 3C, D. The coefficient of determination (R^2^) gives the value of the proximity of the experimental data to the modeled surfaces. An R^2^ value of > 95% was obtained for all the model equations. This observation suggests that the model efficiently represented the actual printing width of the microchannel wall. The predicted values of the two models closely matched the experimental data. The deviation between the predicted dimensions and experimental measurement was in the range of 4.5–4.6%. Thus, the model can be employed to predict the printing outcomes of a microchannel wall. Using this model, the printing parameters can be modified according to the design size of the microchannel wall. Microfluidic devices can be fabricated accurately using this model.

### Characterizations of channel printing accuracy

**Figure 4:**
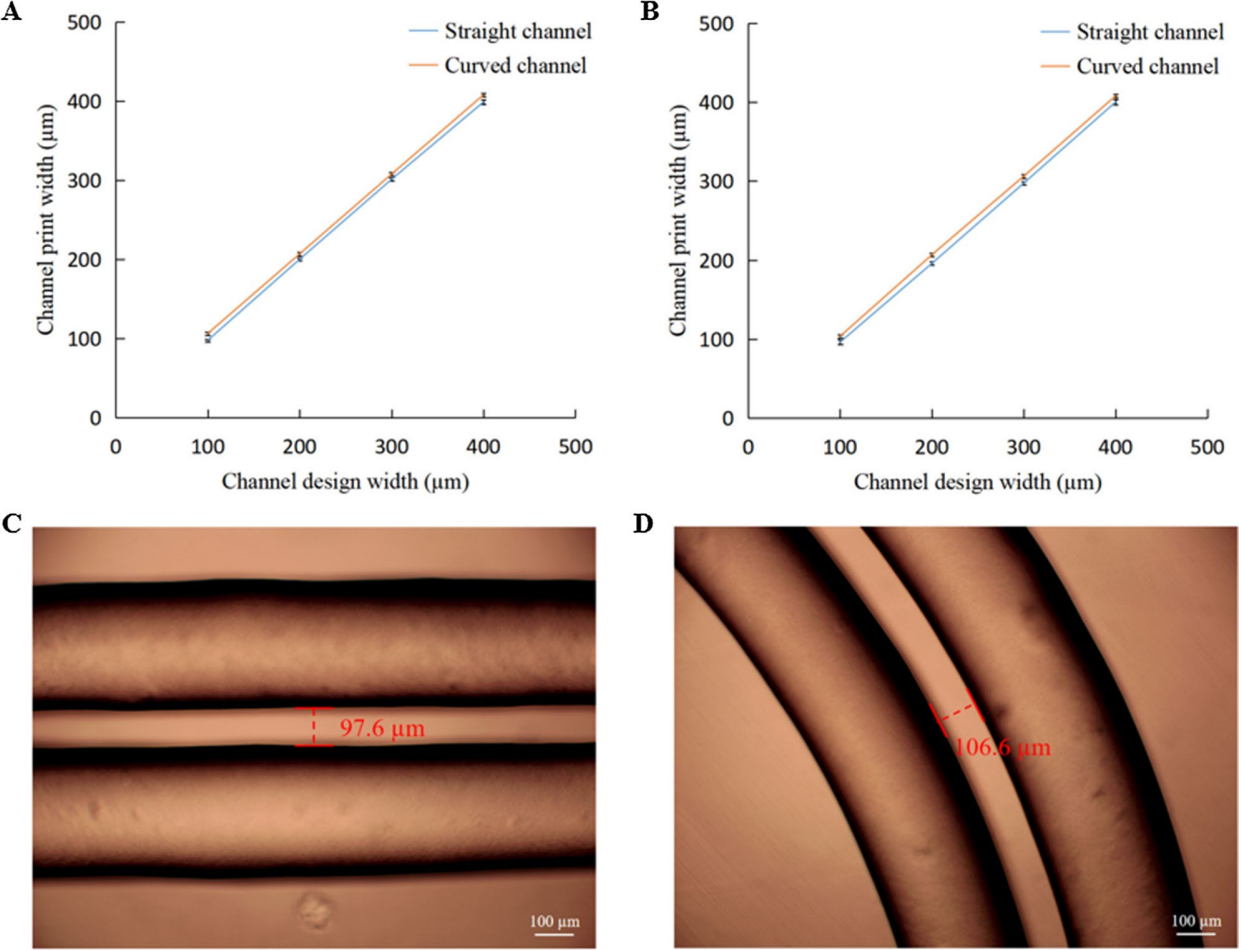
(**A**) Plot of actual print widths of channel against the designed dimension for a 340 µm nozzle, and (**B**) plot of actual print widths of channel against the designed dimension for a 420 µm nozzle (each test channel is printed three times and measured). (**C**) Example diagram of straight channel printed by a 340 µm nozzle, and (**D**) example diagram of curved channel printed by a 340 µm nozzle.

The printing speed was set to 1 mm/s, and the printing pressures were 59 and 58.5 psi, respectively (matched by the optimization model), for the 340 and 420 µm nozzles. The accuracy of the printed channel dimensions was evaluated by printing test channels of 100–400 µm channel width. Figure 4A, B depict the deviations in the dimensions of the printed structures from the designed dimensions for the two types of nozzles. The resulting channel cross sections were further compared with the designed dimensions. The actual printed widths of the straight and curved channels were almost the same as the designed dimensions, with accuracy errors within 10 µm. This small deviation can be explained by the printing setup employed. Owing to the change in the distance from the nozzle to the acrylic substrate, there was a slight difference in the actual printed width of the channel. Moreover, it was observed that the printed channel wall surface was extremely smooth, and the channel wall width also maintained a high fidelity (Fig. 4C, D).

**Figure 5:**
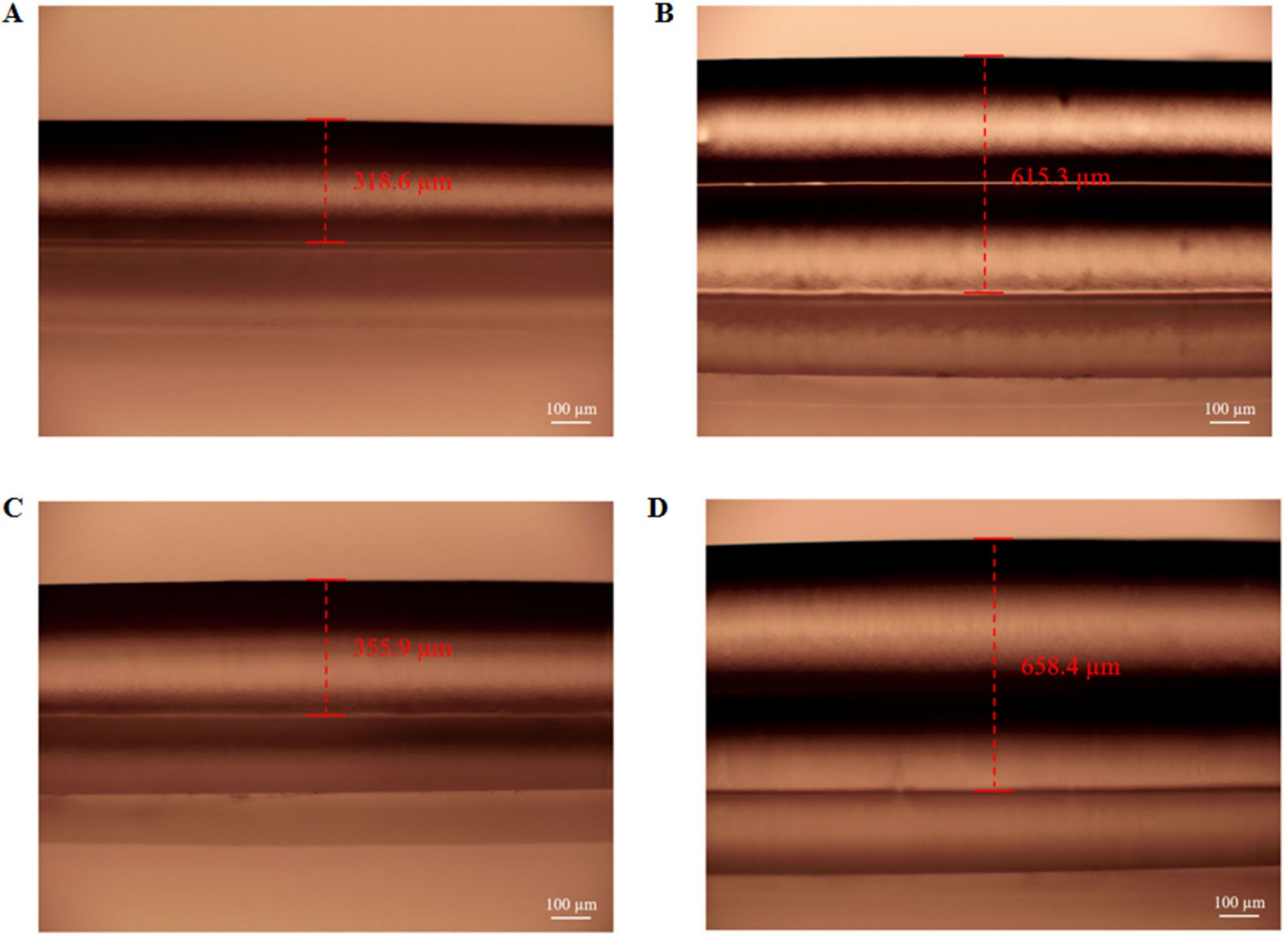
(**A**) Microscope image of single-layer high channel structure printed by a 340 µm nozzle; (**B**) Microscope image of double-layer high channel structure printed by a 340 µm nozzle; (**C**) Microscope image of single-layer high channel structure printed by a 420 µm nozzle; (**D**) Microscope image of double-layer high channel structure printed by a 420 µm nozzle.

The accuracy deviation between the designed channel height and the actual printing height was studied. Figure 5A, B depict the printed average heights of the channel with designed single-layer and double-layer heights of 340 µm nozzles of 318 and 615 µm, respectively (with a height loss of 22 and 65 µm). The channels printed by 420 µm nozzles also had height deviations of 65 and 182 µm, respectively (Fig. 5C, D). In addition, one can clearly observe that the bottom wall was significantly deformed by the downward compression of the top wall (which increased the height loss) for the double-layer structure (Fig. 5B, D). Therefore, high-precision channels can be obtained by printing from small nozzles and by adopting a single-layer wall structure.

### 3D-Printed microfluidic devices

**Figure 6:**
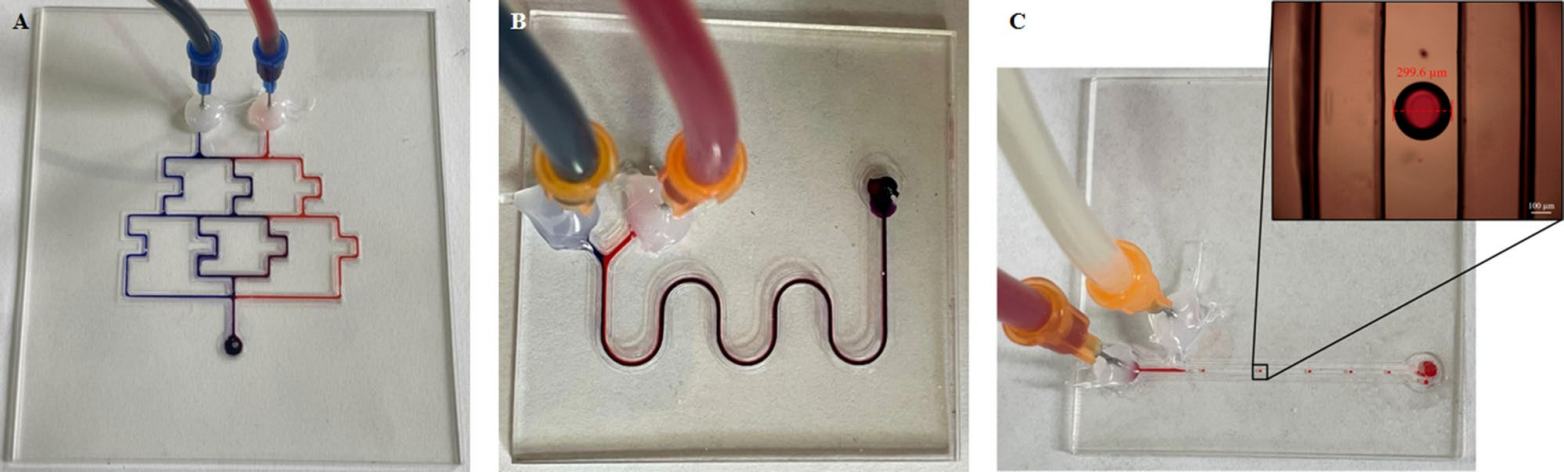
(**A**) Micromixer, (**B**) concentration gradient generator, and (**C**) droplet generator: the above microfluidic devices were printed by 340 µm nozzles.

Several examples of microfluidic devices were printed to demonstrate the versatility of the 3D printing of Dowsil 732 (Fig. 6). To shorten the fabrication time, the printing speed was set to 2 mm/s, and the printing pressure was set to 69 psi (matched by the optimization model). A concentration gradient generator with a channel diameter of 400 µm was printed. It shows effective mixing along the channel cascade, resulting in a color gradient from blue to red (Fig. 6A). A micromixer with a channel width of 400 µm was also printed (Fig. 6B). Figure 6A and B show the mixing process of the blue and red dyes. The blue and red dyes were injected into the micromixer using a two-channel syringe pump (Braintree, MA, USA, Mod. BS-300) actuating on 10 mL disposable syringes. A clear interface appears between the two fluids when first injected. The mixing performance improved when two fluids flowed through the serpentine channel. For a broader application scope, a droplet generator was also printed (Fig. 6C). When red dye and oil were injected into the channel at the flow rate of 0.2 and 1.2 mL/h respectively, the red dye was equidistant cut into multiple droplets with a diameter of 300 µm by the oil. All three microfluidic devices were printed using the optimization model, and the fabrication times were 32, 24, and 22 min, respectively (including the curing time of Dowsil 732).

## Conclusion

A time-efficient and inexpensive fabrication method for transparent 3D-printed microfluidic devices using commercially available silicone rubber as the structural material was reported. The microfluidic devices were designed using SolidWorks, fabricated using a low-cost customized DIW 3D printer, and then capped by a transparent acrylic plate. The printing method proposed in this work is capable of accurately printing 2D (two-dimensional) channel structures. The microfluidic devices were cured for 16 min at a temperature and humidity of 70 °C and 40%, respectively. The surface hydrophobicity of Dowsil 732 were evaluated, proving its suitability for the fabrication of microfluidic devices. To ensure that a high-precision microchannel structure can be obtained, the influences of printing pressure and printing speed on the accuracy of the channel wall were studied, and a printing parameter optimization model was established. The accuracy between the microchannel design size and the actual printed size was evaluated. Three 3D-printed microfluidic devices (a micromixer, concentration gradient generator, and droplet generator) were printed and verified to behave almost as satisfactorily as similarly designed PDMS microfluidic devices. The method of fabricating accurate 3D-printed microfluidic devices with both transparency and time efficiency could make extrusion-based 3D printing a very attractive alternative for the fabrication of microfluidic systems.

## Materials and methods

### 3D-Printing platform

**Figure 7:**
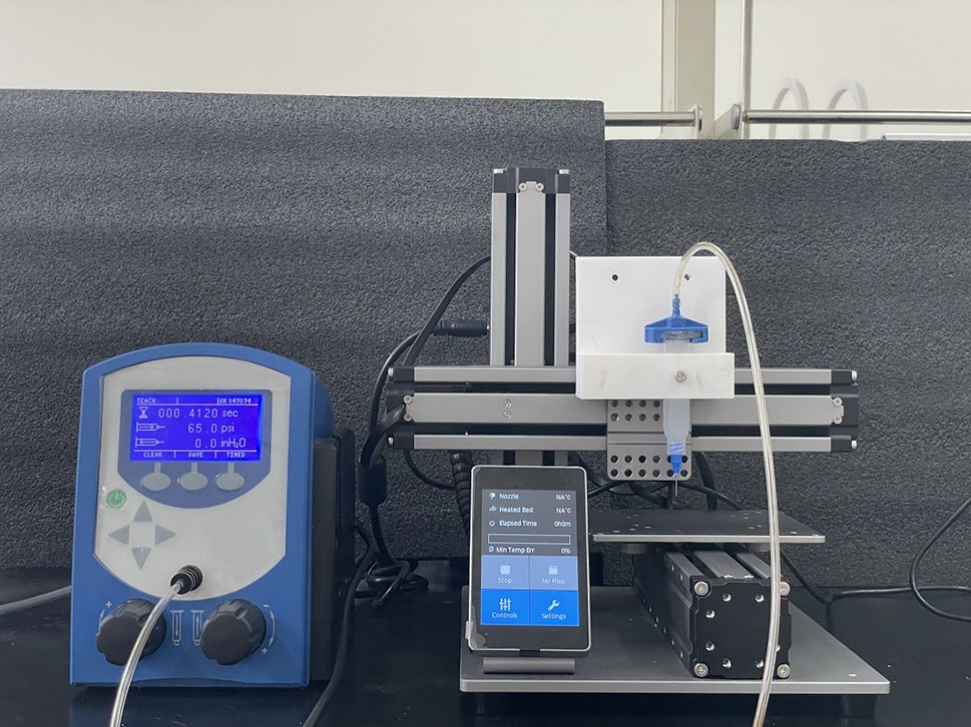
Photograph of customized 3D-printing platform.

The 3D-printing platform is illustrated in Fig. 7. Based on the principle of DIW printing technology, the original thermoplastic extruder of a commercial FDM printer (Snapmaker Original, Snapmaker, China) was replaced with a 3D-printed bracket installed on a 10 mL Luer-Lok syringe (Nordson EFD, USA). The printing material was extruded pneumatically through a Luer-Lok syringe nozzle, which was connected to a pressure regulator (Ultimus I, Nordson EFD, USA). The gas source was supplied by an air compressor (OTS-980, Outstanding, UK). The extrusion speed can be controlled by adjusting the pressure, and the flat-ended Luer-Lok syringe nozzles can be flexibly replaced with different inner diameters according to actual needs.

### Printing strategies and printing materials

**Figure 8:**
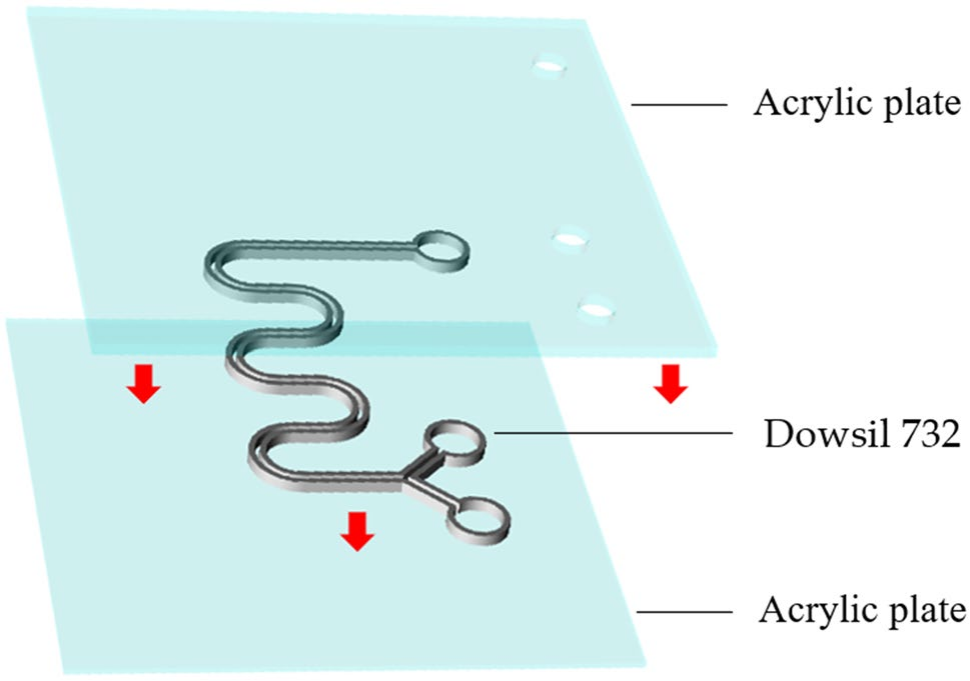
Printing process of microfluidic devices.

The printing strategy is to print the channel pattern directly on the transparent acrylic substrate and cure at room temperature for 5 min. The top plate is then added and a 10 g aluminum plate is placed on its surface to ensure the seal of the channel. Finally, the microfluidic device (Fig. 8) is placed on a heating platform (PC-420D, CORNING, USA) until the printing material is fully cured. This strategy can effectively shorten the fabrication time of microfluidic devices and can also be used with other optical instruments for real-time detection in the channel. All microfluidic device designs are first created using SolidWorks software and converted to the STL format. Simplify 3D software (RepRap, Germany) is used for setting printing parameters and for slicing STL files into G-code.

Dowsil 732 (Dow Corning, Midland, MI, USA), a viscous creamy single-component silicone rubber, is used like PDMS as a silicone sealant or encapsulant in various industries, and it has good resistance to high temperatures (coefficient of volumetric thermal expansion is 1.12 × 10^–3^ 1/K) and has ductility. Dowsil 732 can be directly bonded without any preprocessing for such materials as glass, metal, and plastic. In this work, Dowsil 732 was used as a printing material, and the curing time and hydrophilicity of the material were tested. We printed a 5 mm long Dowsil 732 material using a 420 µm nozzle and took a cross-section in its middle position. The curing time is determined by observing the curing state through a microscope (Fig. S1). In addition, we tested the surface hydrophobicity of Dowsil 732 by depositing a 10 µL drop of DI water mixed with red dye on a 3D printed Dowsil 732 sheet with a pipette. In order to minimize measurement error, the test was repeated at three different locations on the surface tested. A camera (EOS 5D Mark IV, Canon, Japan) was used to record the contact angle pictures and ImageJ (version 1.51j8; NIH; USA) software was used to measure the angles.

### Optimization model of printing parameters

The flat-ended Luer-Lok syringe nozzles (Nordson EFD, USA) with inner diameters of 340 and 420 µm were chosen for microfluidic device fabrication (Fig. S2), and the design widths of the microchannel walls were 340 and 420 µm, respectively. However, the actual widths of the channel walls were affected by the printing pressure and speed during the printing process. To obtain a more accurate microchannel wall structure, a mathematical model with the printing pressure and speed as input, and the microchannel wall width as output, was established. We measured the actual width of the channel walls with and without the top plate (Fig. S3). The accuracy deviation between them is only within 10 µm, so we choose the actual width of the channel walls without the top plate for the printing parameter optimization model. Single walls with uniform length (3 × 10^4^ µm) at different printing speeds (1, 2, 3, 5, and 7 mm/s) and printing pressures (55, 60, 65, and 70 psi) were printed as the modeling samples, and the actual widths of the samples were measured using an optical microscope (MM-8C, Puda, China). The measured data were processed and modeled based on the Sklearn module in Python 3.8, and the accuracy and stability of the optimization model of the printing parameters were evaluated using the coefficient of determination (R^2^).

### Characterization of channel printing accuracy

The performance of the printing parameter optimization model was further evaluated by testing the lateral and vertical printing accuracy of the microchannel. We choose the actual width and height values of the channels without the top plate for the analysis of printing accuracy. Four types of microchannel were designed with different diameters (100, 200, 300, and 400 µm). The actual widths of these channels were measured using the microscope. The vertical printing accuracies of microchannels with different height designs (single-layer and double-layer structures, where the thickness value of each layer depends on the nozzle diameter) were also measured.

## Supplementary Information

Supplementary Figures.

## Data Availability

All data generated or analysed during this study are included in this published article.
