# Supplementary material for: Time-efficient fabrication method for 3D-printed microfluidic devices
Source: Sci Rep. 2022 Jan 24;12:1233. doi: 10.1038/s41598-022-05350-4 (PMC8786882; doi:10.1038/s41598-022-05350-4)
Supplement: Supplementary file 1 — Supplementary Figures. [file 41598_2022_5350_MOESM1_ESM.pdf]

Supplementary Information

Time-efficient fabrication method for 3D-printed microfluidic devices

Yan Jin [1], Peng Xiong [1], Tongyu Xu [1,2,*] and Jingyi Wang [1,2,*]

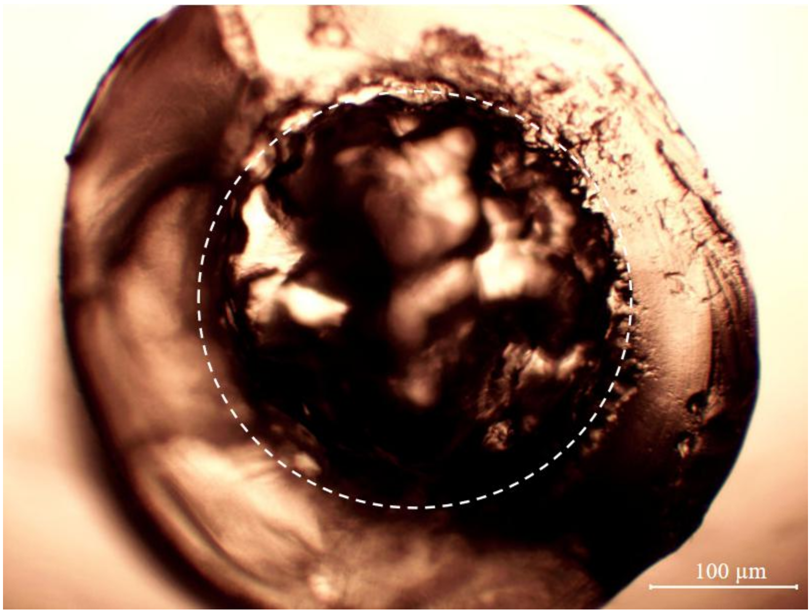

Figure S1. Microscope image of cross-section of printed Dowsil 732. The white dotted line indicates the boundary between cured and uncured material.

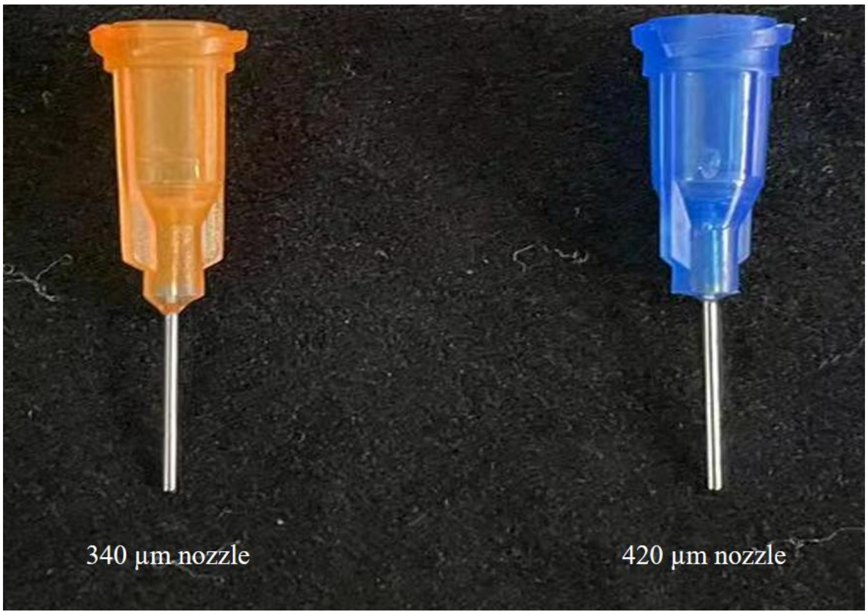

Figure S2. Photograph of Luer-Lok syringe nozzles with different inner diameter sizes.

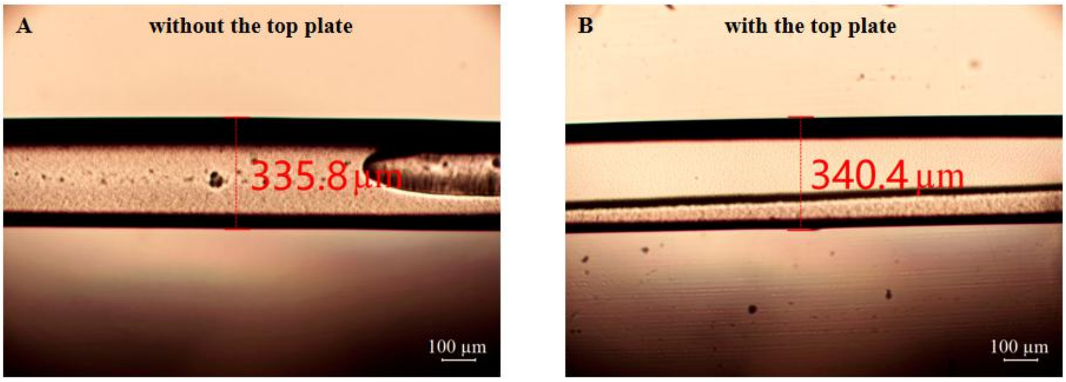

Figure S3. (A) Measurement of the wall width without the top plate; (B) Measurement of the wall width with the top plate.

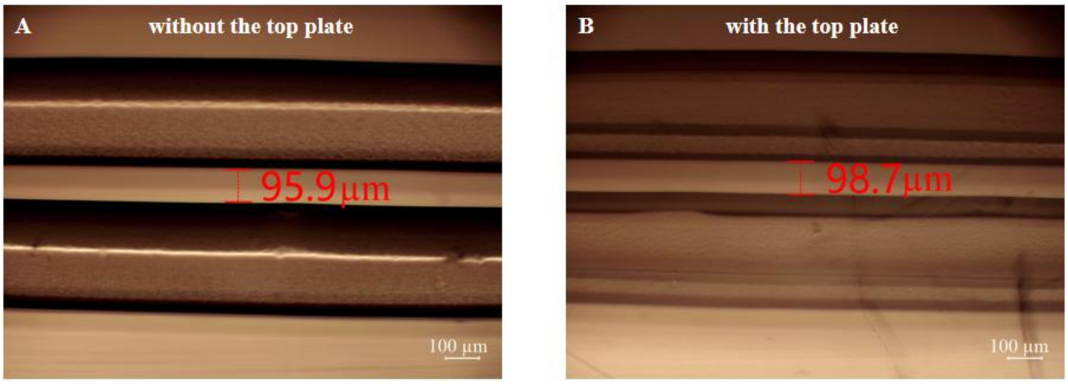

Figure S4. (A) Measurement of the channel width without the top plate; (B) Measurement of the channel width with the top plate.

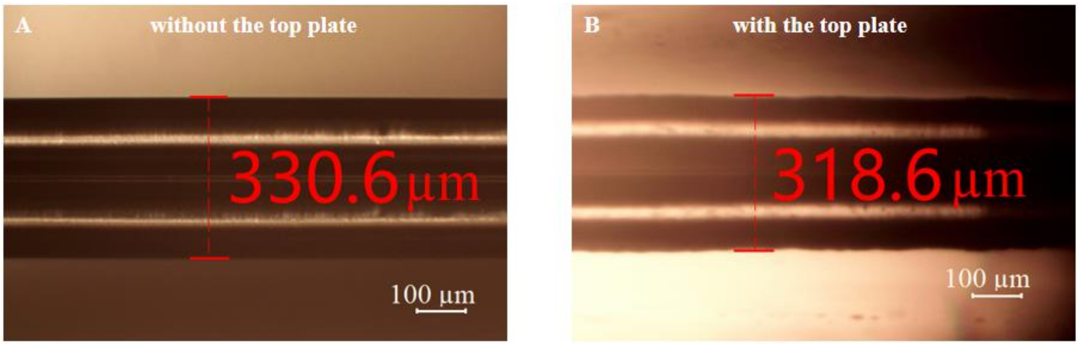

Figure S5. (A) Measurement of the channel height without the top plate; (B) Measurement of the channel height with the top plate.
